# A Web- and App-Based Connected Care Solution for COVID-19 In- and Outpatient Care: Qualitative Study and Application Development

**DOI:** 10.2196/19033

**Published:** 2020-06-01

**Authors:** Timo Schinköthe, Mariano Rolando Gabri, Manfred Mitterer, Pedro Gouveia, Volker Heinemann, Nadia Harbeck, Marion Subklewe

**Affiliations:** 1 CANKADO Cologne Germany; 2 Breast Center Department of Gynecology and Obstetrics University Hospital, Ludwig Maximilian University of Munich Munich Germany; 3 CANKADO Latin America Buenos Aires Argentina; 4 Oncohematological Day Hospital General Hospital Meran Meran Italy; 5 Breast Unit Champalimaud Clinical Center Lisbon Portugal; 6 Department of Medicine III University Hospital, Ludwig Maximilian University of Munich Munich Germany

**Keywords:** COVID-19, eHealth, connected care, telecare, cloud solution, telehealth, public health, infectious disease, pandemic, outbreak

## Abstract

**Background:**

From the perspective of health care professionals, coronavirus disease (COVID-19) brings many challenges as well as opportunities for digital health care. One challenge is that health care professionals are at high risk of infection themselves. Therefore, in-person visits need to be reduced to an absolute minimum. Connected care solutions, including telehealth, remote patient monitoring, and secure communications between clinicians and their patients, may rapidly become the first choice in such public health emergencies.

**Objective:**

The aim of the COVID-19 Caregiver Cockpit (C19CC) was to implement a free-of-charge, web- and app-based tool for patient assessment to assist health care professionals working in the COVID-19 environment.

**Methods:**

Physicians in Argentina, Germany, Iran, Italy, Portugal, Switzerland, and the United States explained their challenges with COVID-19 patient care through unstructured interviews. Based on the collected feedback, the first version of the C19CC was built. In the second round of interviews, the application was presented to physicians, and more feedback was obtained.

**Results:**

Physicians identified a number of different scenarios where telemedicine or connected care solutions could rapidly improve patient care. These scenarios included outpatient care, discharge management, remote tracking of patients with chronic diseases, as well as incorporating infected physicians under quarantine into telehealth services.

**Conclusions:**

The C19CC is the result of an agile and iterative development process that complements the work of physicians. It aims to improve the care and safety of people who are infected by COVID-19.

## Introduction

Health systems around the world face an unprecedented new challenge with the rapid and unexpected spread of coronavirus disease (COVID-19), where limited physical interactions and self-isolation are needed to prevent and reduce infection rates. COVID-19 has become a primary concern for people worldwide. Without the establishment of isolation policies and practices of self-disinfecting and avoidance of interpersonal physical contact, potential infections would have reached 7 billion people, with 40 million deaths around the world [1]. With some exceptions, countries have adopted strict policies so that the expected number of deaths for 2020 will be much less than projected. However, living in these conditions brings forth different levels of discomfort and distress. Half of noninfected persons is feeling a moderate-to-severe psychological impact, and one-third have moderate-to-severe anxiety [2]. Electronic health (eHealth) technology can be a useful tool for supporting the everyday care of patients as well as healthy people. The emergence of COVID-19 in 2019 may, therefore, propel significant steps toward the largescale implementation of digital support in medicine, and experiences of population surveillance and disease monitoring at a population level can be seen.

From the perspective of health care professionals (HCPs), COVID-19 brings many challenges but also rapidly increases the need for digital health care. Because HCPs are also at risk of infection themselves, in-person visits need to be reduced to an absolute minimum. Connected care solutions, including telehealth, remote patient monitoring, and secure communications between clinicians and their patients, may thus rapidly become a significant tool during public health emergencies [3].

Many health centers experience an influx of anxious and infected people. A contact-free prescreening tool is needed to optimize patient control. In another perspective, care management of patients with chronic diseases who are under treatment or observation is also challenging. The risk of getting infected while visiting outpatient departments should be minimized as much as possible.

Another problem is observed in the quickly established departments for COVID-19 care. Under normal circumstances, vital data are usually sent automatically to a centralized monitor; in these units, however, vital data exchange is missing. Since inpatient resources are limited, both early discharge and assurance of patient safety with remote monitoring are needed. Patients can be discharged but need to remain connected via real-time, electronic communication to a remote medical team until full recovery. Another use-case would be physicians who are themselves under COVID-19 quarantine. They cannot do in-person visits, but they can support patients via an easily accessible connected care platform.

The idea of the COVID-19 Caregiver Cockpit (C19CC) was to build a free-of-charge, web- and app-based solution where all these different scenarios (ie, patient screening and visit preparation; remote monitoring; hospital ward cockpit) are supported within a single platform [4].

## Methods

### The COVID-19 Caregiver Cockpit

The C19CC is based on the existing CANKADO environment. The underlying architecture of the multilingual CANKADO is a cloud-based electronic health record (EHR) system with access rights management and function-based access options [5]. Feature packages can be enabled and disabled according to the patient’s illness (eg, diabetes, cancer, etc) and HCP type (eg, oncologist, cardiologist, nurse, psychologist, etc). Information is stored and encoded, allowing physicians and patients to see the EHR in their preferred language.

The CANKADO solution has been developed and operates according to ISO 27001 and ISO 13485. Continuous penetration tests are performed according to the Open Web Application Security Project guidelines.

CANKADO is available through the web or as an app. Patients log in through the CANKADO website or the CANKADO Patient App [6,7] to access their data. HCPs can also use the web access or the HCP Pro App [8,9]. Other connected apps do exist but are not related to the COVID-19 module.

### This Study

Unstructured interviews with physicians in different countries involved in COVID-19 patient care were conducted to identify all the scenarios where telemedicine or connected care solutions could improve patient care. During the first round, physicians in Argentina, Germany, Iran, Italy, Portugal, Switzerland, and the United States explained their challenges with COVID-19 patient care, followed by questions regarding opportunities to improve care using telemedicine or connected care. Based on the feedback collection during round one interviews, the first version of the C19CC was built. In the second round of interviews, the application was presented to the doctors, and their feedback was obtained.

## Results

### Physicians’ Needs

During the first round of interviews, physicians disclosed the following primary needs: an overview of patients housed within provisionary COVID-19 wards, prescreening larger groups of outpatients, keeping close and continuous contact with patients with chronic diseases (mainly cancer patients), improving discharge management, and involving infected physicians in quarantine in patient care.

During the second round of interviews, the most crucial changes requested were to simplify the enrollment process, reduce HCPs’ workload, and implement ways of contact-free interactions between physicians and patients (or separated by a window) with a smartphone app for ward doctors with immediate push notifications. After implementing these additional requests, the application underwent an additional round of review, of which the final results are described in the following sections.

### The Personal Diary

Patients who want to track personal observations that may be related to COVID-19 can use the system as a personal diary. To do so, they have to download the app and select the COVID-19 extension during the registration process.

**Figure 1:**
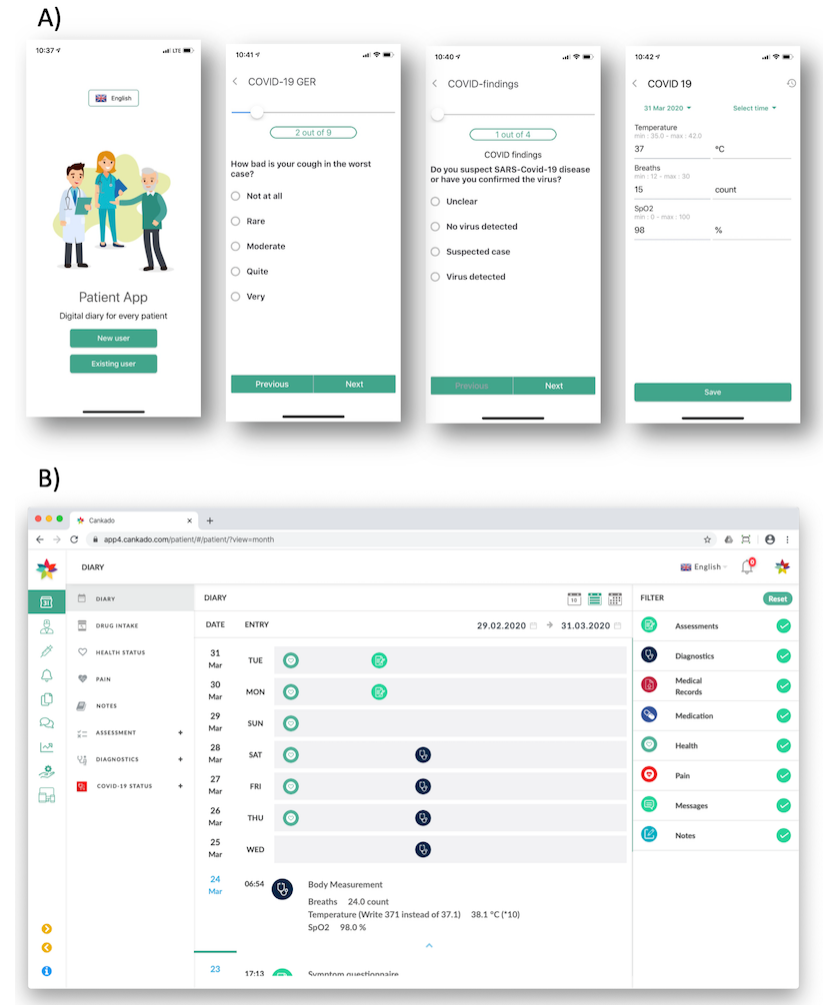
Screenshot examples from the patients’ app for login and data entry (A) and web view of the patient diary (B).

Features are categorized into three groups. After the registration process is completed, patients will receive a questionnaire as a primary assessment to clarify general risk factors and relevant comorbidities. A second questionnaire asks for all cold symptoms according to the validated PRO-CTCAE (Patient Reported Outcomes–Common Terminology Criteria for Adverse Events) questions [10]. This questionnaire is triggered once daily to ensure regular updates from the patient. A third questionnaire is for continuously tracking necessary vital parameters like body temperature and respiratory rate. For those who have a pulse oximeter, oxygen saturation can also be documented. Furthermore, other COVID-19 findings can also be reported via the system (Figure 1).

### The Health Care Professional Cockpit

**Figure 2:**
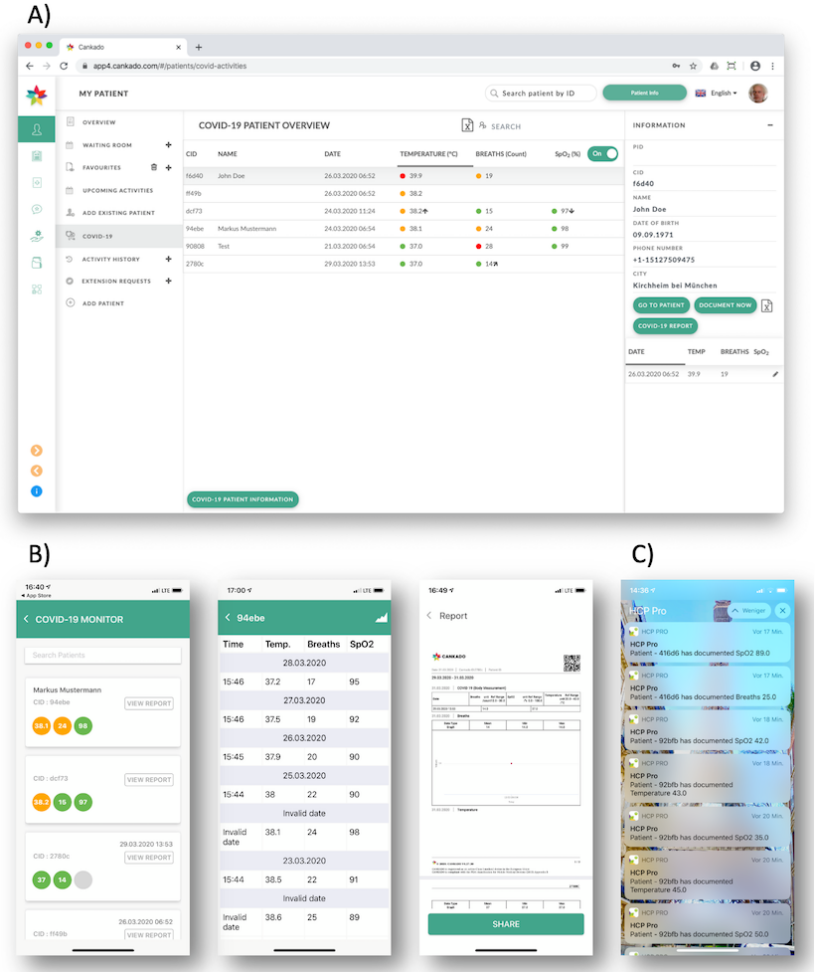
The COVID-19 cockpit view for health care professionals (A); patient list, patient details, and a PDF report preview using the HCP Pro App (B); push notification alerts for health care professionals if a patient’s condition deteriorates (C).

If patients and HCPs are connected, the HCPs can see all their COVID-19 patients in a separate cockpit. A fast and accessible overview of all patients is the main intention of the system. The central window provides the patient list with the latest vital parameters, color coded for severity, with arrows indicating changes compared to the day before. Two export features allow data transfer either from the patient list or from an individual patient history into a table format. The “COVID-19 Report” generates a single PDF file with the entire COVID-19-related history of a patient, and the “COVID-19 Patient Information” button creates a printout for easy patient linkage (Figure 2).

### Contact-Free Linkage

The system provides several ways to link or connect patients and doctors without having physical contact. The connecting process can be initiated by both the patient and the doctor.

**Figure 3:**
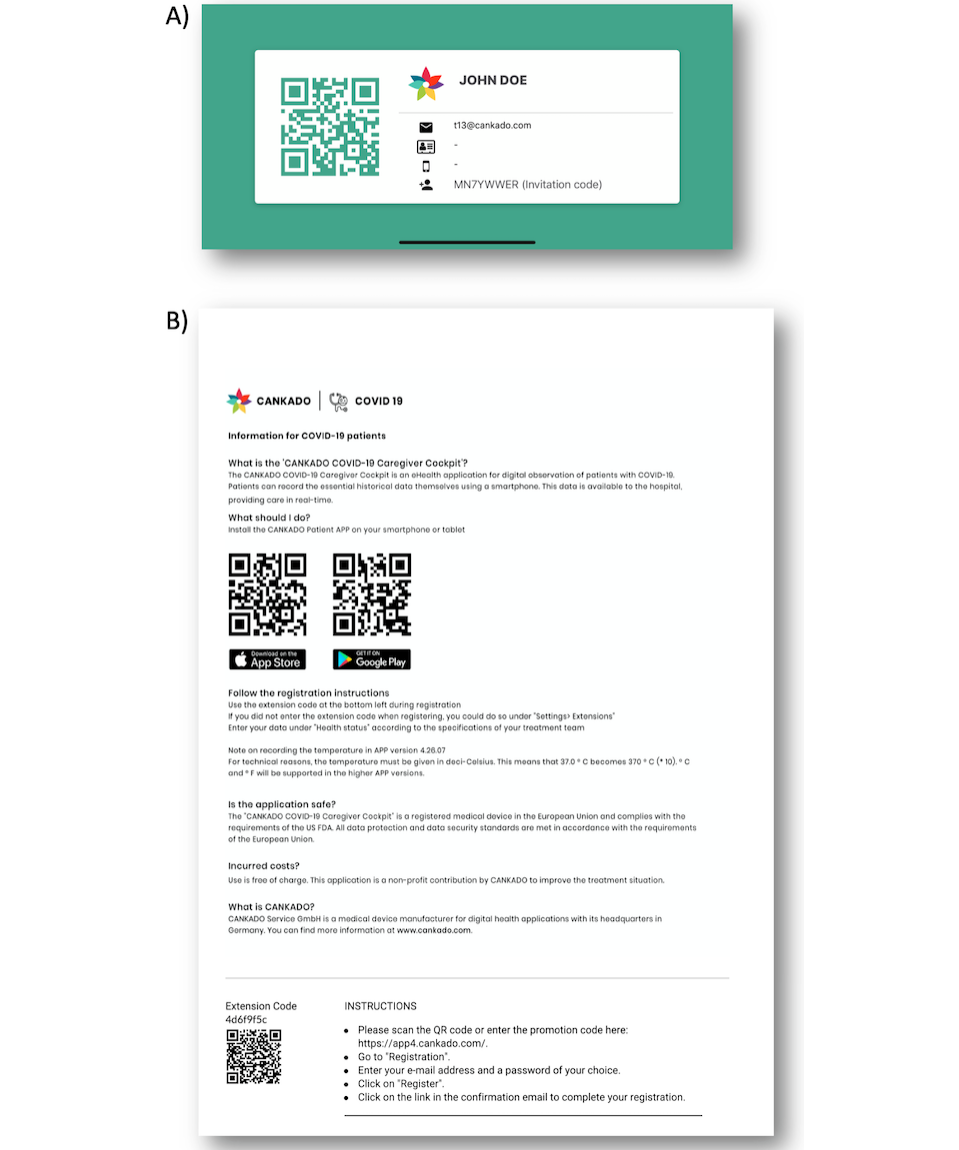
Invitation code using the patient app for a contact-free meeting with their physician (A); patient instruction for self-linking to a center (B).

Patients have three options for connecting with their physicians. After launching the app, the patient can rotate the smartphone into landscape orientation. The screen automatically switches to an identifier page like a business card (Figure 3A). This identifier contains a QR (Quick Response) code for direct scanning and invitation code. The QR code can be scanned by the physician through the HCP Pro App. This scanning procedure can also be performed through a closed glass door. The invitation code is intended for transmission by telephone. To do this, the doctor must select the function “Add Existing Patient” in the web portal. For those patients who prefer not to use the app, an invitation letter can be generated in the web portal by selecting the “Invite Physician” feature. This function creates a PDF document that is intended for use via fax, email, or regular mail.

To connect the other way around, physicians can generate a patient information page by selecting “COVID-19 Patient Information.” This printout contains instructions for patients as well as a center-specific extension code (Figure 3B). This extension code can be used for an unlimited number of patients. Patients who are using this code will receive the COVID-19 extension and are automatically connected to the health center that generated the printout.

### Scenario 1: Patient Screening and Visit Preparation

Once a patient is linked to a health center and has completed the assessments, all information can be printed at once using the “COVID-19 Report” feature in the cockpit view. Within a single click, a PDF is generated, which contains all COVID-19-related information, including a graphical view of vital parameters. To link patients with their HCP, all previously described methods can be used.

### Scenario 2: Remote Monitoring

Remote monitoring is intended for several use-cases. For example, patients can continue to be observed after discharge, or physicians who are under quarantine can take care of their patients remotely. For this purpose, the C19CC provides real-time access to all documented data. In case data are asked and provided via phone, doctors have the chance to enter the data immediately; should errors occur on the patient's side, the data can be edited.

### Scenario 3: Use in a Hospital Ward

The provisional COVID-19 wards cannot often monitor patient data centrally. In these situations, the C19CC, in combination with the HCP Pro App for physicians, can be used. If a patient documents worsening vital parameters, connected doctors immediately get a push notification via the app and can review the patient’s history (Figure 2C). The web view also supports real-time monitoring.

## Discussion

The C19CC is the connected care, solution-driven result of a joint international collaboration between physicians who are taking care of COVID-19 patients. It includes several scenarios in routine care.

The most critical use-cases are undoubtedly in the outpatient departments, which are overrun by patients. Here, the application helps to prescreen patients in a contact-free manner and to get a fast overview of those patients who most urgently need help. Improving workflows and reducing workload in provisional COVID-19 wards constitute another vital application to help relieve some of the overburdened human resources while ensuring patient safety at the same time.

As of February 20, 2020, 20% of all HCPs in Italy taking care of COVID-19 patients have become infected themselves [11]. Physicians who are infected or who had close contact with infected persons have to remain under quarantine. However, these medical resources often remain unused during the quarantine period. Enabling these doctors to take care of patients remotely helps to keep them integrated with the delivery of medical care in a time when resources are scarce.

One very vulnerable group is patients with chronic diseases. Cancer patients, in particular, are at increased risk for severe events compared to noncancer patients [12]. The same seems to apply to patients suffering from hypertension, diabetes mellitus, fatty liver/abnormal liver function, chronic gastritis/gastric ulcer, coronary heart disease, hyperlipidemia, cholelithiasis, arrhythmia, thyroid diseases, electrolyte imbalance, urolithiasis, stroke, chronic renal insufficiency, aorta sclerosis, secondary pulmonary tuberculosis, or chronic obstructive pulmonary disease [13]. These patients should only go to the outpatient clinic if it is unavoidable in order to minimize their risk of infection. The described system can now support their care by real-time, electronic communication between a patient and their physician, including telehealth, remote patient monitoring, and secure communication between clinicians and their patients.

In conclusion, the C19CC demonstrates how eHealth technology can quickly adapt to actual changing needs in the health care environment and implement a system that can aid HCPs in patient care and ensure patient safety at the same time. The C19CC is registered as an active medical device in the European Union and compliant with the FDA classification for Mobile Medical Devices (2015) Appendix B.

## Abbreviations

C19CC: COVID-19 Caregiver Cockpit
COVID-19: coronavirus disease
eHealth: electronic health
EHR: electronic health record
HCP: health care professional
PRO-CTCAE: Patient Reported Outcomes–Common Terminology Criteria for Adverse Events
QR: Quick Response
